# High-grade squamous intraepithelial lesion arising adjacent to vulvar lymphangioma circumscriptum: a tertiary institutional experience

**DOI:** 10.18632/oncotarget.10158

**Published:** 2016-06-17

**Authors:** Go Eun Bae, Gun Yoon, Yong Jung Song, Hyun-Soo Kim

**Affiliations:** ^1^ Department of Pathology, Graduate School, Kyung Hee University, Seoul, Republic of Korea; ^2^ Department of Obstetrics and Gynecology, Pusan National University Yangsan Hospital, Pusan National University School of Medicine, Yangsan-si, Gyeongsangnam-do, Republic of Korea; ^3^ Department of Pathology, Severance Hospital, Yonsei University College of Medicine, Seoul, Republic of Korea

**Keywords:** lymphangioma circumscriptum, vulva, high-grade squamous intraepithelial lesion, human papillomavirus

## Abstract

Lymphangioma circumscriptum of the vulva occurs in patients who have undergone radical hysterectomy, lymph node dissection, or radiation therapy for management of advanced uterine cancer. Since vulvar lymphangioma circumscriptum typically presents as multiple, grossly verrucous vesicles of various sizes, it may be impossible to clinically distinguish vulvar lymphangioma circumscriptum from other vulvoperineal cutaneous diseases. In the present study, 16 (1.6%) out of the 1,024 vulvar biopsy or excision specimens were diagnosed as lymphangioma circumscriptum. In two (12.5%) out of the 16 cases, unusual histopathological findings were observed. Both patients had previously undergone radical hysterectomy with lymph node dissection and postoperative radiation therapy or concurrent chemoradiation therapy for advanced cervical cancer. Microscopic examination revealed high-grade squamous intraepithelial lesions, which were located immediately adjacent to the normal squamous epithelium covering the dilated subepithelial lymphatic vessels. Further, human papillomavirus genotyping confirmed that both patients were infected with high-risk human papillomavirus. High-grade squamous intraepithelial lesion cannot be grossly distinguished from vulvar lymphangioma circumscriptum because the multiple, verrucous vesicles that constitute the characteristic gross appearance of vulvar lymphangioma circumscriptum hinder its distinction. In this regard, our cases of high-grade squamous intraepithelial lesion, located adjacent to vulvar lymphangioma circumscriptum, support the notion that active surgical excision is necessary for the treatment of vulvar lymphangioma circumscriptum.

## INTRODUCTION

Lymphangioma circumscriptum (LC) is a hamartomatous lymphatic malformation of the lymphatic channels of the skin, typically associated with a developmental anomaly of the lymphatics [1]. It presents as a relatively well-circumscribed, localized lesion consisting of small, grouped vesicles that can ooze and drain lymphatic and sanguineous fluids. The vesicles cause significant morbidity and psychological distress from symptoms including itching, pain, oozing, and secondary infections. Although the diagnosis of LC is most often established by physical examination and clinical inspection, biopsies or surgical excisions followed by histopathological examination can help differentiate LC from other cutaneous vesicular diseases, such as herpesvirus infections, dermatitis herpetiformis, contact dermatitis, metastatic carcinoma, grouped or eruptive pyogenic granulomas, hemangiomas, or malignant melanomas. Imaging studies, using ultrasonography, computed tomography, and/or magnetic resonance imaging, can also help to determine the extent of the lymphatic cisterns [2, 3].

High-grade squamous intraepithelial lesions (HSILs) are squamous lesions that carry significant risk of development of invasive cancer, if not treated. HSILs cannot be grossly observed during routine clinical examination, except when they are exophytic or papillary, and they are not conspicuous on colposcopy. We recently experienced two HSIL cases, arising adjacent to vulvar LC, which occurred following surgery and postoperative concurrent chemoradiation therapy (CCRT) or radiation therapy (RT) for advanced cervical cancer. The possibility of HSIL was not considered during the physical examination of patients, and vulvar LC lesions, which had increased in size and caused symptoms, were completely excised. Histopathologically, the HSILs were found incidentally and were located adjacent to the LC lesions. Because a HSIL cannot be identified grossly when it arises adjacent to vulvar LC, it may be overlooked due to the vulvar LC-specific gross findings (multiple, conglomerated, verrucous vesicles). Our experience serves to remind the pathologists and gynecologists that HSILs can arise adjacent to vulvar LC and that checking for the presence of grossly unidentifiable HSIL by completely excising the vulvar LC lesion is important when LC develops in patients who have a history of surgery, radiation therapy, or concurrent chemoradiation therapy for advanced uterine cancer.

## RESULTS

### Patient demographics

During the period from January 2000 to December 2015, 1,024 patients underwent either biopsies or excisions for benign or malignant vulvar lesions. The age of patients ranged between 10 and 83 years (median, 45 years). The pathological diagnoses in 1,024 patients with vulvar lesions are shown in Table 1. In addition, the clinicopathological profiles of 16 patients diagnosed with vulvar LC are summarized in Table 2. Nine out of the 16 patients had a previous history of gynecological cancer. Six patients had cervical cancer, and each of the remaining three patients had cancers of the endometrium, vagina, and vulva, respectively. Surgical treatment included radical abdominal hysterectomy with unilateral or bilateral salpingo-oophorectomy and pelvic lymph node dissection and total abdominal hysterectomy for cervical cancer, and wide local excision for vulvar cancer. Nonsurgical treatment included CCRT for endometrial and cervical cancers and RT for cervical and vaginal cancers. The International Federation of Gynecology and Obstetrics (FIGO) stages in these nine patients were IA1 in one patient, IB in one patient, IIB in three patients, IIIB in one patient, and IIIC in one patient. The FIGO stages of the remaining two patients were not available. Since these nine patients with gynecological cancers received either surgery, CCRT, or RT, all of them were considered to have developed the acquired type of vulvar LC. The median age of patients at presentation was 55 years (range, 43–81 years). The mean interval between cancer treatment and the development of vulvar LC was 11.3 years (range, 5–20 years). In two out of the six patients with previous cervical cancer, high-risk human papillomavirus (HPV) was detected in the vulvar LC lesions, whereas HPV was not detected in the remaining four patients.

**Table 1:** Pathological diagnosis in 1,024 patients who had undergone vulvar biopsy or excision

Category	Pathological diagnosis	Number of cases
Malignant	Squamous cell carcinoma	56
	Extramammary Paget disease	55
	Bowen disease	44
	Basal cell carcinoma	16
	Malignant melanoma	16
	Sarcoma	11
	Metastatic carcinoma	11
Precancerous	High-grade squamous intraepithelial lesion (VIN 3)	38
	High-grade squamous intraepithelial lesion (VIN 2)	32
Benign: Neoplastic	Lymphangioma circumscriptum	16
	Lipoma/fibrolipoma	11
	Syringoma	10
	Hidradenoma papilliferum	10
	Dermatofibroma	8
	Squamous papilloma	7
	Angiomyofibroblastoma	4
	Other	9
Benign: Nonneoplastic	Chronic nonspecific vulvitis	152
	Cyst*	112
	Condyloma acuminatum	79
	Lichen sclerosus	59
	Fibroepithelial stromal polyp	42
	Benign epidermal lesion**	33
	Nevus	18
	Ulcer	17
	Dermatitis***	15
	Herpesvirus infection	9
	Other	93
No pathological abnormality		41
Total		1,024

**Abbreviations:** VIN: vulvar intraepithelial neoplasia;

* Cyst includes Bartholin gland cyst, mucinous cyst, Mullerian cyst, and epidermoid cyst.

** Benign epidermal lesion includes seborrheic keratosis, hyperkeratosis, parakeratosis, and acanthosis;

*** Dermatitis includes chronic eczematous dermatitis, interface dermatitis, and superficial perivascular dermatitis.

**Table 2:** Clinicopathological profiles of 16 patients with vulvar lymphangioma circumscriptum

Case	Age at diagnosis	Previous history of gynecological cancer	Treatment	Interval between cancer treatment and LC diagnosis	LC recurrence	Association with HSIL	HPV status	Current status
1	10	None	NA	NA	NA	No	NA	LTF
2	21	None	NA	NA	NA	No	NA	LTF
3	27	None	NA	NA	NA	No	NA	NED
4	29	None	NA	NA	NA	No	NA	LTF
5	32	None	NA	NA	NA	No	NA	LTF
6	55	None	NA	NA	NA	No	NA	LTF
7	61	None	NA	NA	NA	No	NA	LTF
8	55	Em EC, IIIC	CCRT	5 years	Yes (thrice)	No	NA	NED
9	43	Cx SCC, IA1	RAH+RSO+BPLND	11 years	Yes (thrice)	No	Not detected	NED
10	46	Cx SCC, IIB	CCRT	5 years	No	No	Not detected	NED
11	49	Cx SCC, IIB	RAH+BSO+BPLND+RT	8 years	No	Yes	HR HPV detected*	NED
12	65	Cx SCC, IIIB	RAH+BSO+BPLND+CCRT	16 years	No	Yes	HR HPV detected**	NED
13	70	Cx SCC	TAH+RT	20 years	Yes (thrice)	No	Not detected	NED
14	81	Cx SCC, IIB	RT	19 years	No	No	Not detected	DOO
15	46	Vag HGNEC	RT	7 years	Yes (twice)	No	NA	AWD
16	57	Vul BCC, IB	WLE+RT	11 years	Yes (twice)	No	NA	NED

**Abbreviations:** AWD: alive with disease, BPLND: bilateral pelvic lymph node dissection, BSO: bilateral salpingo-oophorectomy, CCRT: concurrent chemoradiation therapy, Cx SCC: cervical squamous cell carcinoma, DOO: dead of other condition, Em EC: endometrial endometrioid carcinoma, HR HPV: high-risk HPV, HSIL: high-grade squamous intraepithelial lesion, LC: lymphangioma circumscriptum, LTF: lost to follow-up, NED: no evidence of disease, NA: not applicable, RAH: radical abdominal hysterectomy, RSO: right salpingo-oophorectomy, RT: radiation therapy, TAH: total abdominal hysterectomy, Vag HGNEC: vaginal high-grade neuroendocrine carcinoma, Vul BCC: vulvar basal cell carcinoma, WLE: wide local excision;

* Type 16;

** Types 33 and 56.

### Clinical findings

HSILs were found in two (12.5%) of the 16 patients diagnosed with vulvar LC. Neither patient had a history of congenital LC. Each of the two patients had undergone surgery and CCRT and RT, respectively, after being diagnosed with advanced cervical cancer. Furthermore, neither patient had a history of vulvar HSILs or vulvar cancer before cervical cancer treatment.

Patient 1 was a 65-year-old woman who presented with edema and bilateral labia majora papules that oozed clear fluid. Sixteen years earlier, the patient had undergone a radical hysterectomy, pelvic lymph node dissection, and subsequent CCRT for FIGO stage III cervical squamous cell carcinoma. Although a few papules appeared, for the first time, one year prior to her current admission, they were left untreated. These papules had recently increased in size and number and were accompanied by edema and oozing. The papules were initially diagnosed, at a private clinic, as genital warts and were treated with podophyllotoxin. However, the symptoms did not improve, and the patient was transferred to our institution. Upon examination, multiple papules with verrucous surfaces were noted on bilateral labia majora. Considering the patient's medical history, we surgically excised the lesions due to the possibility of LC. The postoperative course was uneventful, and she was discharged from the hospital 5 days after surgery. No recurrence was apparent over the last 8 months. The patient is alive with no evidence of disease.

Patient 2 was a 49-year-old woman who presented with edema and erythema of the right labium majora. Some vesicles, oozing clear fluid, were also noted on the vulva. Eight years earlier, she had undergone a radical hysterectomy with pelvic lymph node dissection for FIGO stage IIB cervical squamous cell carcinoma, followed by RT. Although carbon dioxide laser vaporization was performed to treat the right vulvar lesions, they increased in number and the area of skin involved continued to expand. Upon examination, multiple, variable-sized papules with verrucous surfaces were noted on the right labium majora; in a few areas, conglomerates were observed. Complete surgical excision was performed. The postoperative course was uneventful, and the patient left the hospital 4 days later. The patient remained well without specific events or complications for 1 year postoperatively.

### Histopathological and immunohistochemical findings

**Figure 1:**
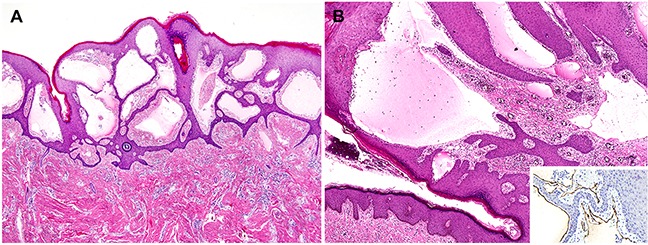
Histopathological findings of vulvar lymphangioma circumscriptum **A.** The upper dermis shows irregularly shaped, cystically dilated lumina. The overlying epidermis varies in thickness. Over some of the cystic spaces, the epidermis is thinned, but elsewhere it shows acanthosis, hyperkeratosis, and irregular down-growth, enclosing some of the dilated lymphatic channels. **B.** These lymphatic channels contain eosinophilic material and a few lymphocytes and erythrocytes. A single layer of bland-looking endothelial cells are highlighted by immunostaining for the lymphatic endothelial cell marker D2-40 (inset).

Microscopic examination of the vulvar LC lesions revealed multiple, irregular-shaped, variable-sized lumina containing eosinophilic material in the upper dermis (Figure 1A). Some of the channels were cystically dilated. The overlying epidermis showed a few areas of mild pseudoepitheliomatous hyperplasia. The channels were lined by a single layer of bland endothelial cells highlighted by immunostaining for the lymphatic endothelial cell marker D2-40 (Figure 1B). In twelve out of the 16 cases with vulvar LC, a variable degree of hyperkeratosis was noted. The upper dermal chronic inflammatory infiltrate was associated with LC in five cases. In three cases, basal hyperpigmentation was identified adjacent to LC. As a separate lesion, condyloma acuminatum was observed in a single case.

**Figure 2:**
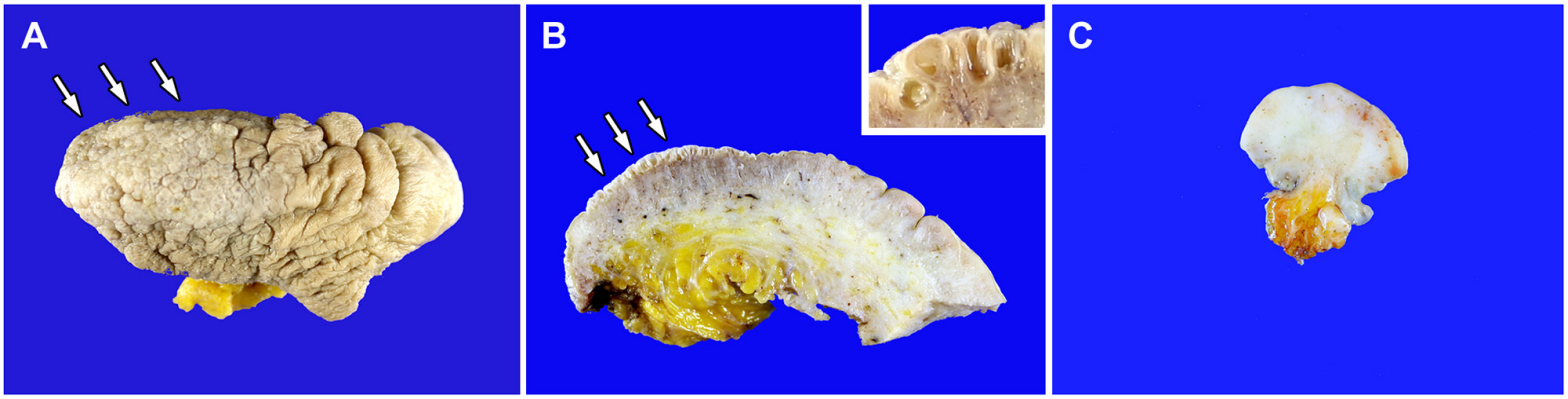
Gross findings **A.** Case 1. The resected specimen reveals a hyperpigmented, rugose surface, with multiple, conglomerated papules (white arrows). **B.** Case 1. The cut section shows small, papillary projections (white arrows) with thin-walled cystic cavities (inset) confined within the upper dermis, characteristic of lymphangioma circumscriptum. **C.** Case 2. The cut section shows fibrotic dermis, without a mass-like lesion. The superficial dermis displays some punctate hemorrhagic spots, but there is no grossly evident cystic lesion. In neither case were any gross findings, indicative of high-grade squamous intraepithelial lesion, observed.

Gross examination of the resected specimens of Case 1 revealed hyperpigmented, rugose, and studded epidermis with multiple conglomerated papules, measuring up to 1–7 mm (Figure 2A). The cut sections showed nodularity and papillary projections with thin-walled cystic cavities in the superficial dermis. The deep dermis showed a few areas of mild fibrotic changes, and the subcutaneous tissue was unremarkable (Figure 2B). The specimen of Case 2 showed a gray-to-white, fibrotic cut surface, without a mass-like lesion (Figure 2C). The superficial dermis displayed some punctate hemorrhagic spots, but unlike Case 1, no grossly clear, thin-walled cystic lesions were observed.

**Figure 3:**
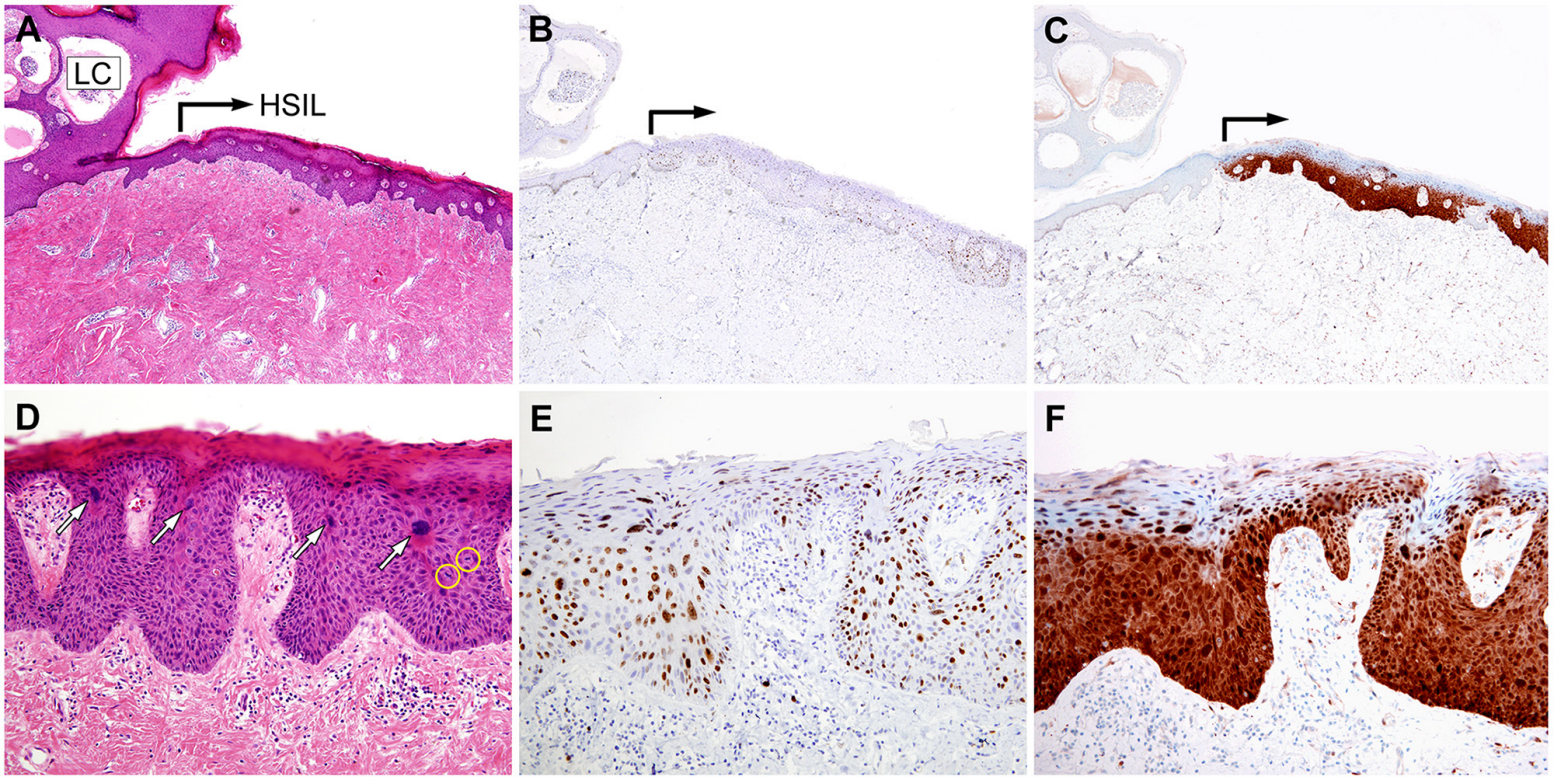
Histopathological and immunohistochemical findings of Case 1 **A.** The right upper corner shows cisterns of lymphatic channels, partially enclosed by irregularly growing epidermis (LC). High-grade squamous intraepithelial lesion (HSIL) is located approximately 1 mm away from the lymphangioma circumscriptum (right-angled black arrow). **B.** and **C.** The atypical squamous epithelium exhibits strong nuclear immunoreactivity for (B) Ki-67 and (C) p16, confirming the HSIL diagnosis. **D.** High-power view of image A. Significant nuclear pleomorphism (white arrows) and frequent mitotic figures (yellow circles) are noted. No evidence of stromal invasion, indicative of invasive squamous cell carcinoma, is evident. **E.** High-power view of Image B. The Ki-67-positive atypical nuclei reside within the entire thickness of the squamous epithelium. **F.** The continuous, strong nuclear p16 immunoreactivity, i.e., p16 block positivity, indicates HSIL.

Histopathological examination of Case 1 sections revealed multiple dilated channels in the papillary dermis, containing eosinophilic, proteinaceous material and a few erythrocytes, consistent with LC (Figure 3A). Proliferation of the neoplastic squamous epithelium, displaying abnormal nuclear features (including significantly increased nuclear size, irregular nuclear membranes, and an increased nucleo-cytoplasmic ratio accompanied by mitotic figures) was observed 1 mm away from the lesion. Obvious nuclear pleomorphism, frequent mitotic figures in the middle and upper thirds of the epithelium, and the presence of atypical mitoses were diagnostic of HSIL [vulvar intraepithelial neoplasia (VIN) 3; Figure 3D]. The HSIL exhibited a significantly increased Ki-67 labeling index (Figure 3B and 3E) and block positivity for p16 (Figure 3C and 3F); no invasion or desmoplastic stromal response was observed.

**Figure 4:**
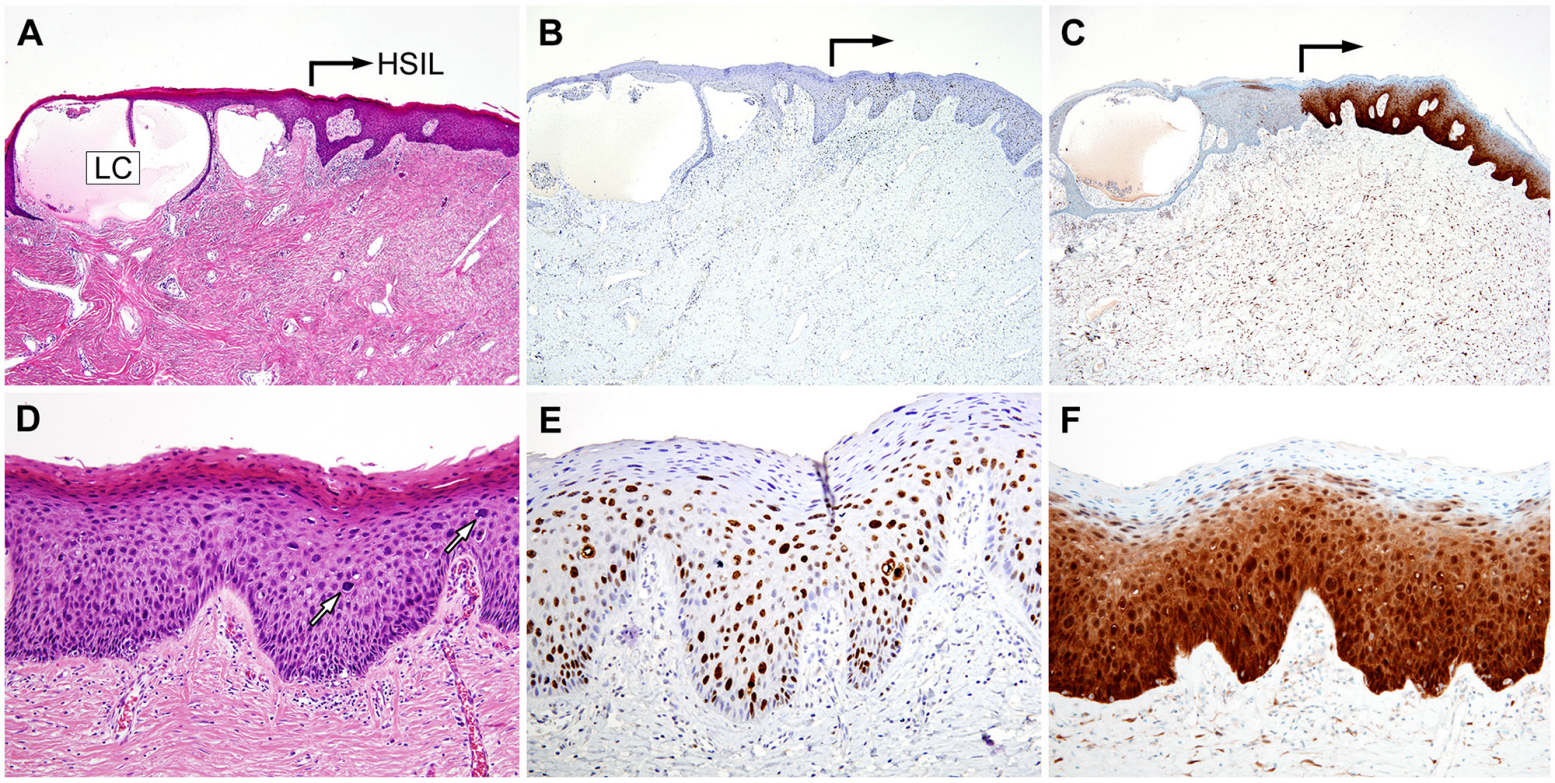
Histopathological and immunohistochemical findings of Case 2 **A.** A few cystically dilated lymphatic channels are partially enclosed by thin cords of squamous epithelium, extending from the epidermis (LC). High-grade squamous intraepithelial lesion (HSIL) is continuous with the epidermis that overlies the lymphangioma circumscriptum (right-angled black arrow). **B.** and **C.** The HSIL exhibits (B) a significantly increased Ki-67 labeling index and (C) strong nuclear p16 immunoreactivity. **D.** High-power view of Image A. The white arrows indicate enlarged, pleomorphic nuclei. No evidence of stromal invasion is observed. **E.** High-power view of Image B. The large, atypical nuclei in the lower and middle thirds of the epithelium display strong Ki-67 immunoreactivity. **F.** The p16 block positivity confirms the HSIL diagnosis.

Similar histopathologic findings were also observed in Case 2. Cystically dilated lymphatic channels and focal pseudoepitheliomatous hyperplasia of the epidermis were diagnostic of LC (Figure 4A). A HSIL (VIN 3) was found immediately adjacent to this lesion (Figure 4D). The Ki-67 labeling index significantly increased in the HSIL, compared to the non-neoplastic squamous epithelium (Figure 4B and 4E), and the lesion showed block positivity for p16 (Figure 4C and 4F); no invasion or desmoplastic stromal response was observed.

**Figure 5:**
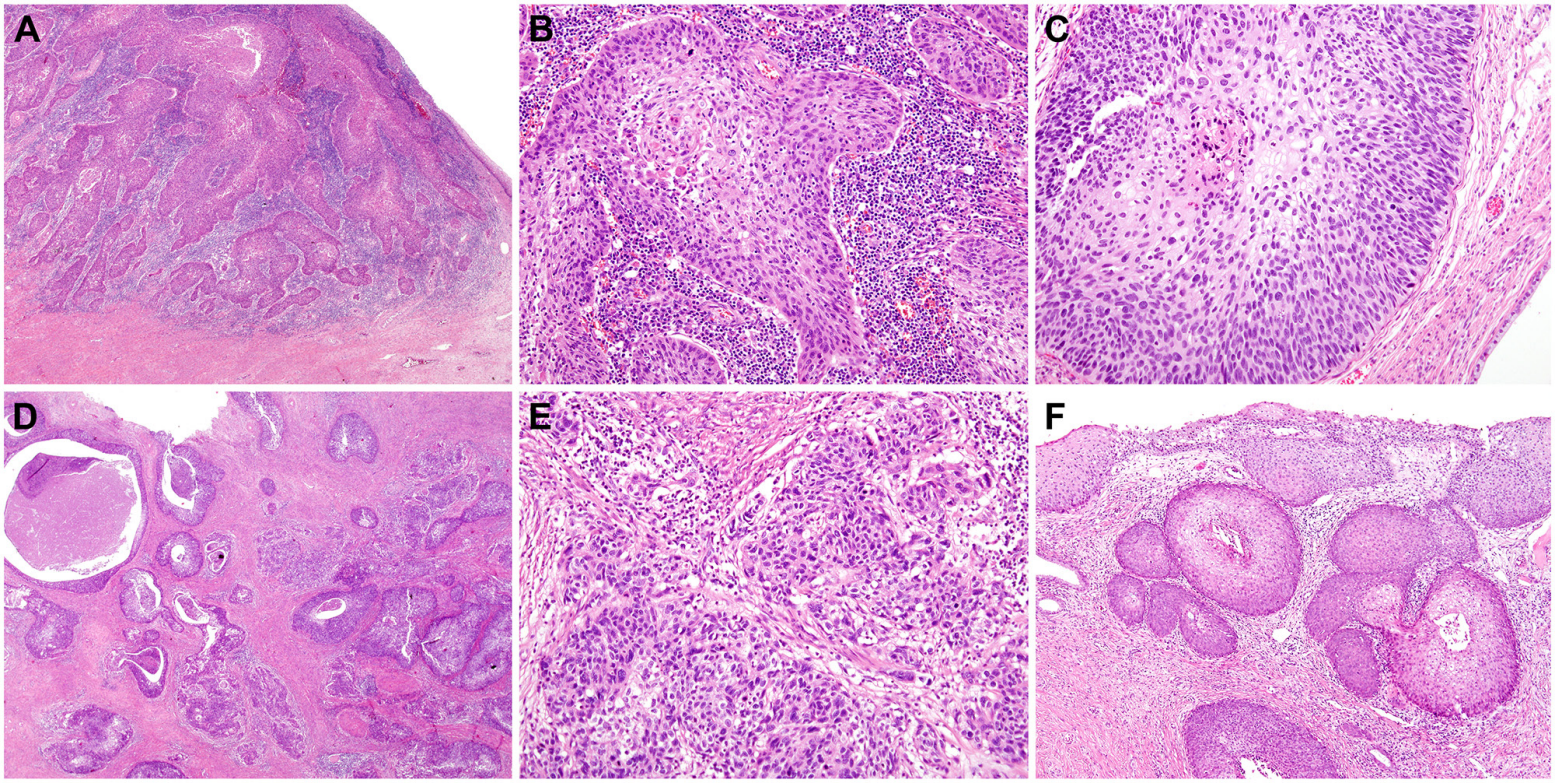
Histopathological findings of previous cervical cancer: (A to C) Case 1 **A.** A low-power view of cervical invasive squamous cell carcinoma. **B.** A medium-power view shows irregular-shaped tumor cell sheets and dense inflammatory infiltrate in the stroma. Some dyskeratotic cells are also noted. **C.** A medium-power view of cervical HSIL involving the endocervical gland. (D to F) Case 2. **D.** Variable-sized sheets and nests of tumor cells are randomly distributed and deeply infiltrate the cervical stroma. **E.** Infiltrating tumor cells are embedded in desmoplastic stroma. **F.** In the peritumoral areas, cervical HSIL exhibits an extensive endocervical glandular extension.

We compared the histopathological features between previous cervical cancer and vulvar HSILs. Histopathological examination of cervical cancer tissue sections revealed characteristic morphological features of invasive squamous cell carcinoma in both cases (Figure 5). Areas of HSIL background showed focal keratinization at the surface, scattered dyskeratotic cells and occasional squamous pearl formation. Compared to cervical HSILs, the vulvar lesions showed some distinctive morphological features: a thick layer of keratinization on the surface and markedly pleomorphic and severely atypical cells scattered throughout the entire epithelial thickness. Based on these unique features, the vulvar lesions could be classified as keratinizing HSILs in both cases.

### Human papillomavirus genotyping results

**Figure 6:**
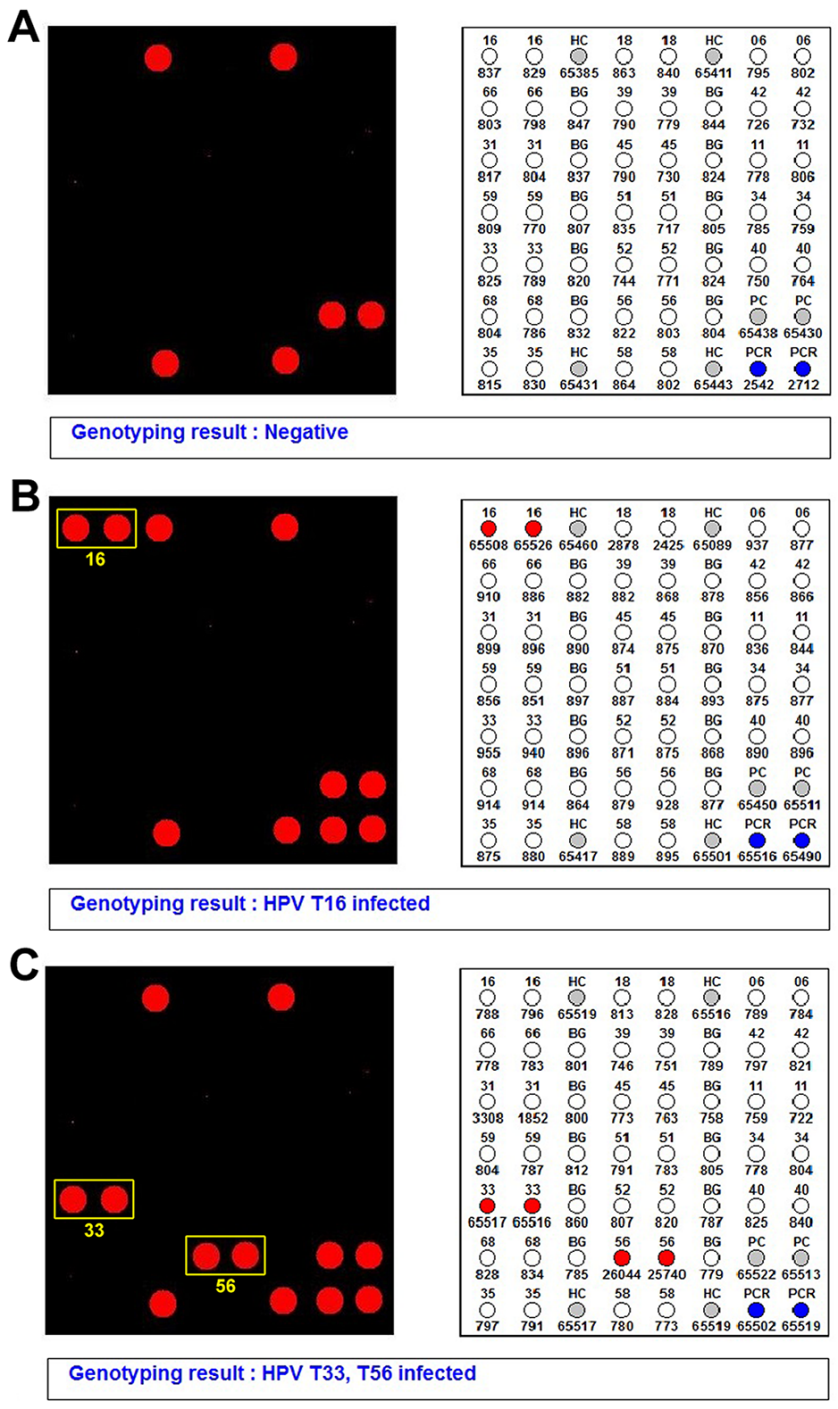
HPV genotyping results **A.** The vulvar lymphangioma circumscriptum unrelated to HSIL does not exhibit HPV infection. **B.** In Case 1, the 9G DNA Chip detects high-risk HPV infection (type 16; yellow box). **C.** In Case 2, the vulvar lesion was found to be co-infected with high-risk HPV types 33 and 56 (yellow boxes).

HPV DNA was not detected in the LC lesions that were not associated with HSIL (Figure 6A). In Case 1, the 9G DNA chip detected high-risk HPV (type 16; Figure 6B); in Case 2 also, high-risk HPV (types 33 and 56) was detected (Figure 6C). In both cases, the same HPV genotype was detected in cervical cancers and in vulvar HSILs.

## DISCUSSION

LC occurs in the lymphatic vascular system of the dermis [4]; however, its pathogenesis is unclear. Even though primary vulvar LC can occur congenitally due to a developmental defect in the vulvar lymphatic system, it is a very rare condition. Vulvar LC can also be acquired by uterine cancer patients who have undergone radical hysterectomy, pelvic lymph node dissection, and/or pelvic radiation therapy that damages the lymphatic vasculature [4–9]. Contrary to the uncertainty of vulvar LC pathophysiology, almost all of the vulvar premalignant lesions are associated with HPV infection [10–12]. The incidence of vulvar HSILs has been increasing among young women [11, 13]. Several epidemiologic studies have reported that women diagnosed with cervical HSILs or cancer have higher risk of developing vulvar HSILs or cancers than normal women [14]. This increased risk is most likely due to the oncogenic HPV genotype that causes cervical HSILs and also infects the vulva. As in cervical HSILs, the most important factor causing vulvar HSILs is infection with a high-risk HPV. In the present study, through polymerase chain reaction (PCR)-based HPV genotyping method, we confirmed that vulvar HSILs, which occurred in patients who had been surgically treated for cervical cancer, were caused by the same HPV genotype that caused cervical cancers. We performed a literature search regarding the association of HSIL with vulvar LC; a thorough Medline search, using the PubMed retrieval service, was performed using the key words ‘high-grade squamous intraepithelial lesion’, ‘vulvar intraepithelial neoplasia’, ‘lymphangioma circumscriptum’, ‘lymphangioma’, ‘lymphedema’, and ‘vulva’. However, no reported cases of HSIL arising adjacent to vulvar LC lesions were found, indicating that LC-associated HSIL is quite a rare entity.

The clinical manifestations of vulvar LC include multiple hyperpigmented, rugose papules, which are similar to those observed in other vulvoperineal diseases [15, 16]. Even though vulvar HSILs may appear macular, papular, or condylomatous [10], they cannot be detected grossly unless they are exophytic or papillary. Moreover, it is even more difficult to grossly distinguish small areas of vulvar HSILs located adjacent to LC lesions from LC lesions; as a result, there is a very high possibility that they cannot be detected using a punch biopsy. Although histopathological confirmation with a biopsy or excision is essential to diagnose an HSIL located adjacent to LC, a standardized biopsy protocol has not yet been established because LC-associated HSIL is a very unusual condition.

At present, there is no consensus regarding the standard treatment for vulvar LC. Treatment modalities, reported in the literature, include surgical excision, abrasive methods (carbon dioxide laser, liquid nitrogen, electrocoagulation, or sclerosing therapy), and observation [4, 17–21]. In our previous study that analyzed the clinical outcomes of acquired vulvar LC lesions, we found that surgical excision is helpful for improving symptoms and preventing recurrence [4]. Although nonsurgical options have been attempted to prevent surgery-related complications, and some of these options have been reported to be effective in controlling symptoms, a thorough literature search revealed negative results from these options [17, 18, 20–23]. Cryotherapy seems to be rather ineffective, with low remission and high recurrence rates. Sclerotherapy agents have a potential risk of severe systemic, local, and cosmetic side effects. There are no available data regarding the effectiveness of local therapy for vulvar LC lesions using a large-scale patient cohort. Therefore, local therapy has not been proven to reliably improve patient symptoms, such as pain and/or pruritus. Our previous study demonstrated that surgical treatment options may be favorably considered when the patient fulfills the following criteria: (1) a large mass and deep vulvar LC lesions; (2) the presence of distressing symptoms, such as pain, pruritus, edema, discharge, and secondary infection; and (3) failure of nonsurgical treatment [4]. Our discovery, in this study, that vulvar HSILs can occur adjacent to LC lesions that develop following cervical cancer surgery is significant because it supports the notion that surgical treatment of LC lesions is necessary. We suggest that the most appropriate way to diagnose a HSIL, accompanying an LC, is to increase the possibility of detection through multiple biopsies or by complete excision of the LC lesion.

In summary, we demonstrated that HSILs can occur adjacent to vulvar LC lesions. Further, the same HPV genotypes were found to be associated with both cervical cancers and vulvar HSILs, in patients who had undergone surgical cervical cancer treatment. Gross distinction between HSILs and vulvar LC lesions is almost impossible when both appear simultaneously. Our observation regarding the occurrence of HSILs adjacent to vulvar LC lesions suggests that active surgical excision of vulvar LC is necessary.

## MATERIALS AND METHODS

### Case selection

The cases were selected from the surgical pathology files of Severance Hospital, Yonsei University College of Medicine. During the period from January 2000 to December 2015, 1,024 patients underwent either vulvar biopsies (668 cases; 65.2%) or excisions (356 cases; 34.8%). Of these, 16 (1.6%) patients were diagnosed as having vulvar LC. This study was reviewed and approved by the Institutional Review Board at Severance Hospital, Yonsei University Health System, Seoul, Republic of Korea (2016-0264-001).

### Histopathological examination

The biopsied or resected specimens were fixed in 10% neutral-buffered formalin and embedded in paraffin blocks. From each formalin-fixed, paraffin-embedded (FFPE) block, 4-μm sections were cut, stained with hematoxylin and eosin, and prepared for immunohistochemical staining.

### Immunohistochemistry

FFPE sections were deparaffinized and rehydrated with a xylene and alcohol solution. Immunohistochemical staining was performed using a Ventana Benchmark XT automated staining system (Ventana Medical Systems, Tucson, AZ, USA) or a Dako Omnis System (Dako, Agilent Technologies, Carpinteria, CA, USA), according to the manufacturer's instructions. Antigen retrieval was performed using Cell Conditioning Solution (CC1; Ventana Medical Systems) or EnVision FLEX Target Retrieval Solution, High pH (Dako, Agilent Technologies). Sections were incubated with primary antibodies against D2-40 (1:100, clone D2-40, Dako), p16 (prediluted, clone E6H4, Ventana Medical Systems), and Ki-67 (1:150. clone MIB-1, Dako). After chromogenic visualization, using ultraView Universal DAB Detection Kit (Ventana Medical Systems) or EnVision FLEX /HRP (Dako, Agilent Technologies), slides were counterstained with hematoxylin. Appropriate positive and negative controls were concurrently stained to validate the staining method.

### Human papillomavirus genotype assay

We used PCR-based microarray methods for HPV genotyping, using a commercially available HPV 9G DNA chip (BMT HPV 9G DNA Chip; Biometrix Technology, Chuncheon, Republic of Korea). The 9G test examined the presence of 14 high-risk (16, 18, 31, 33, 35, 39, 45, 51, 52, 56, 58, 59, 66, and 68) and 5 low-risk (6, 11, 34, 40, and 42) HPV types; analyses were performed according to the manufacturer's instructions [22]. Briefly, the PCR mixture consisted of 10 μL of the extracted target DNA, 10 μL of the primer set (provided by the manufacturer), and PCR premix (provided by the manufacturer) that contained dNTP and Taq DNA polymerase in an amplification buffer. Amplification was performed using the following steps: predenaturation for 5 min at 94°C; 40, 30-s denaturation cycles at 94°C; 40, 30-s annealing cycles at 45°C; 40, 30-s elongation cycles at 72°C; and a final 5-min elongation step at 72°C. The PCR products were electrophoresed in a 2% agarose gel to confirm successful amplification. Each hybridization chamber of the 9G DNA chip was covered with a mixture of the hybridization solution (35 μL) and the PCR product (15 μL) and incubated at 23–26°C for 30 min. After washing, array images were scanned and imaged using a fluorescent scanner (ScanArray GX Microarray Scanner, PerkinElmer Life and Analytical Sciences, Waltham, MA, USA).
